# Use of the VELYS Robotic‐Assisted Solution in knee arthroplasty: A scoping review

**DOI:** 10.1002/jeo2.70726

**Published:** 2026-04-20

**Authors:** Scott R. Morrison, Andrew J. Hall, Nick D. Clement, Phil J. Walmsley, Christopher Gee, Jon V. Clarke

**Affiliations:** ^1^ Department of Orthopaedics Golden Jubilee University National Hospital Clydebank UK; ^2^ Scottish Centres for Orthopaedic Treatment and Innovation in Surgery and Healthcare (SCOTTISH) Network Golden Jubilee University National Hospital Clydebank UK; ^3^ School of Medicine University of St Andrews St Andrews UK; ^4^ Edinburgh Orthopaedics, Royal Infirmary of Edinburgh Edinburgh UK; ^5^ Department of Trauma and Orthopaedic Surgery Victoria Hospital Kirkcaldy UK; ^6^ University of Strathclyde Glasgow UK

**Keywords:** arthroplasty, imageless, knee, robotic, VELYS

## Abstract

**Purpose:**

Robotic knee arthroplasty has been associated with improved knee‐specific outcomes, but results are relatively immature. The VELYS Robotic‐Assisted Solution is an imageless semi‐active system used with the ATTUNE knee arthroplasty system. Recently, the UK National Institute for Health and Care Excellence called for a multidomain evaluation of six robotic‐surgery platforms in orthopaedics, of which VELYS is one. This review aimed to (i) evaluate current evidence on VELYS in total knee arthroplasty, (ii) assess outcomes, complications, cost and versatility, (iii) appraise study quality and (iv) identify knowledge gaps.

**Methods:**

A scoping review using five‐stage methodology following Preferred Reporting Items for Systematic Reviews and Meta‐Analyses extension for Scoping Reviews (PRISMA‐ScR) guidelines was undertaken. Articles were screened against pre‐determined criteria, with data synthesized descriptively and thematically.

**Results:**

One hundred twenty‐six studies were identified, with 22 included. Evidence level ranged from II to IV. Analysis highlighted improved implant positioning as seen with other robotic systems, as well as a non‐inferior safety profile. However, despite studies appearing to highlight favourable early patient‐reported outcomes and function as well as greater workflow efficiency, the overall quality of the published work was poor, giving little evidence to robustly support many of the conclusions drawn in these studies. Limitations of these studies included small sample sizes, a lack of information on patient characteristics and patient selection, retrospective design and a lack of long‐term follow‐up.

**Conclusion:**

The available literature regarding the VELYS Robotic‐Assisted Solution is limited and of moderate‐to‐poor quality. Implant positioning was more accurate; however, other results, especially regarding improved patient outcomes, are not currently well‐evidenced. Evidence was largely retrospective or early prospective, with no randomized controlled trials or long‐term data. High‐quality, randomized studies are required to evidence this technology.

**Level of Evidence:**

N/A.

**Abbreviations**
CT: computed tomography
FJS: Forgotten Joint Score
HKA: hip–knee–ankle
KOOS: Knee Injury and Osteoarthritis Outcome Score
MINORS: Methodological Index for Non‐Randomized Studies
OKS: Oxford Knee Score
PRISMA‐ScR: Preferred Reporting Items for Systematic Reviews and Meta‐Analyses extension for Scoping Reviews
PROM: patient‐reported outcome measure
TKA: total knee arthroplasty
VRAS: VELYS Robotic‐Assisted Solution

## INTRODUCTION

Knee arthroplasty is a commonly performed orthopaedic procedure and is a well‐established intervention for the management of end‐stage osteoarthritis [20]. The volume of knee arthroplasty being carried out is projected to continue to increase in the United Kingdom from over 100,000 joints per year currently to almost 140,000 per year by 2060 [35]. Despite the volume of procedures carried out, a proportion of patients are dissatisfied post‐operatively, with recent reviews quantifying as being around 10% of patients after total knee arthroplasty (TKA) [15]. In addition, there remains a growing burden of revision arthroplasty, reflecting the fact that patients are living longer following their index procedure and having higher demands in later life [5]. Robotic and navigation‐based arthroplasty systems have been developed to improve satisfaction and reduce long‐term revision risk [38, 46, 49]. It has been suggested that this technology can yield better functional and implant survivorship outcomes by facilitating greater resection accuracy and preservation of bone, optimized implant positioning and more physiological balancing of the joint [2, 40].

Robotic arthroplasty systems can be defined as passive, where the robot provides positioning of cutting guides under surgeon supervision, semi‐active, where the robot can guide the actions of the surgeon, and provide feedback boundaries for certain actions, or active, where the robot performs bone resection independently [60]. These can be further categorized as image‐based or imageless systems depending on the requirement for preoperative imaging (usually a computed tomography [CT] scan) [23]. Most data on robotic arthroplasty exist regarding semi‐active image‐based systems, primarily Mako SmartRobotics (Stryker); however, there is growing evidence in the field of imageless robotic arthroplasty [41]. The main imageless robotic systems in the imageless space are the ROSA (Zimmer‐Biomet), CORI (Smith + Nephew), ApolloKnee System (Corin) and the VELYS Robotic‐Assisted Solution (Johnson & Johnson MedTech) [41]. The ROSA and ApolloKnee systems are both passive systems that utilize mobile robotic jigs and/or non‐robotic jigs, which are tightened under software guidance. Each of these systems involves using a normal bone saw with no additional feedback during cuts [41]. The CORI system is a semi‐active system that does not involve a robotic arm, but instead relies on a tracked handheld burr for bony resection with image‐based feedback [41]. The VELYS Robotic‐Assisted Solution (VRAS) is an imageless, semi‐active robotic system that employs a robotic arm to guide cuts with a tracked bone saw, while providing image‐based feedback on resection and a real‐time evaluation of joint balance [17]. It is a relatively new addition to the market, receiving U.S. Food and Drug Administration (FDA) 501k clearance in January 2021 [51].

There are studies that have compared image‐based and imageless systems, but informative evaluations are difficult to conduct due to the heterogeneity of hardware, software and functional principles employed by each [23, 57]. Consequently, independent analysis of each system may provide reliable information to guide practice, as reflected by the UK National Institute for Health and Care Excellence (NICE), which called for a multidomain evaluation of the six leading platforms [41].

More research is required to evaluate this developing technological ecosystem in general and, in line with the NICE EVA priorities, the VRAS system specifically [41]. A structured scoping review was carried out in line with Preferred Reporting Items for Systematic Reviews and Meta‐Analyses extension for Scoping Reviews (PRISMA‐ScR) guidance and current best practice for this technique [4, 32, 39, 55]. The objectives were to (i) evaluate the current information available regarding use of the VELYS device in knee arthroplasty, (ii) evaluate the effect of VELYS use in knee arthroplasty on outcomes, complications, cost and versatility, (iii) assess the quality of the evidence available and (iv) identify current knowledge gaps and research priorities.

## METHODS

### Protocol

This scoping review was designed to align strictly with an established five‐stage scoping review methodology following the PRISMA‐ScR and was conducted in accordance with current best practice guidance for this scientific method of literature evaluation [4, 32, 39, 55]. The protocol was constructed following a systematic approach in collaboration with the research stakeholders. The protocol is available upon request from the corresponding author.

### Research questions

i. What literature exists currently reporting on the use of VRAS in knee arthroplasty?
ii. What is the effect of VRAS use in knee arthroplasty on outcomes, complications, cost and versatility?
iii. What is the quality of evidence in the literature?
iv. What are the current evidence gaps and research priorities for this topic?

### Eligibility criteria

All papers that considered VRAS for use in TKA were considered for inclusion. In line with scoping review methodology, any literature on the topic may be assessed; however, to increase the reliability of the evaluated material, inclusion was limited to published articles that used the following robust study designs: structured peer‐reviewed original research, systematic reviews and meta‐analyses, as well as unpublished research theses that used these methodologies [4, 32]. There were no restrictions on the demographics of cases reviewed, so long as the study involved review of VRAS in a primary TKA setting. A publication timeframe from January 2020 to January 2026 (when the search was undertaken on 1 February 2026) was utilized, to include 1 year prior to the commercial introduction of the device to the present day. Only papers published in the English language were reviewed. Exclusions were studies related to hip arthroplasty or trauma, reports where the full text was unavailable, opinion or comment pieces, as well as reports without objective data or empirical findings.

### Information sources and search strategy

An initial search of the literature using terms ‘VELYS’ OR ‘VRAS’ OR ‘Imageless’ OR ‘Image‐free’ AND ‘arthroplasty’ was conducted. Between all databases, including grey literature, this returned over 10,000 results. Through iterative searches and discussion amongst the study group, the final search terms of ‘VELYS’ OR ‘Image‐free robotic orthopaedics’ were used to search through titles, abstracts and keywords of published articles. The search strategy was applied to the following databases to extract relevant literature: PubMed, Directory of Open Access Journals, Web of Science, Scopus, ClinicalTrials.gov and ProQuest Central. Regarding the exploration of the grey literature, Google Scholar was utilized to search for relevant articles. The reference lists of all included studies were screened for any additional relevant articles, and discussions were undertaken with experts on VELYS and its associated research, which identified two further studies.

### Study selection

**Figure 1:**
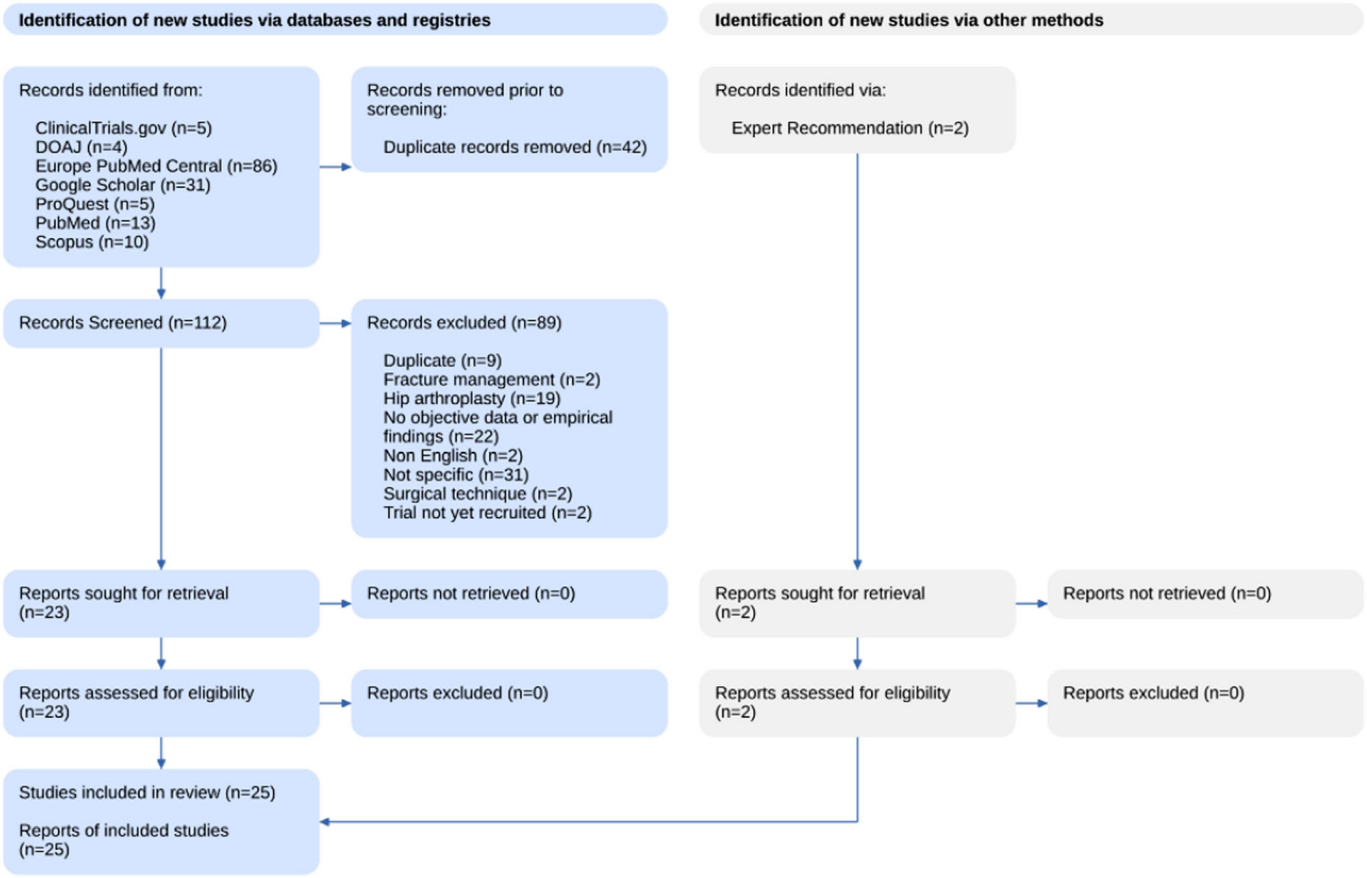
PRISMA‐ScR flow chart of the search process and included articles. PRISMA‐ScR, Preferred Reporting Items for Systematic Reviews and Meta‐Analyses extension for Scoping Reviews.

Following a search of the relevant databases, all identified articles were collated using a dedicated collaborative review platform (Nested Knowledge) and duplicates subsequently removed. Titles and abstracts were screened by two reviewers independently and assessed against the inclusion and exclusion criteria. Full‐text articles were retrieved for all potentially relevant sources, and a detailed assessment against the inclusion and exclusion criteria was conducted for every paper by two reviewers again. No disagreements between the two reviewers occurred; thus, no further discussions with a third reviewer were deemed necessary. The results of this search process were reported using a PRISMA‐ScR flow diagram (Figure 1).

### Extracting the data

The search results were incorporated into data extraction tables to facilitate the extraction of relevant data by the lead reviewer. A secondary reviewer reviewed all included papers to extract data into identical tables to ensure accuracy of extraction and agreement between reviewers. There were no disagreements in data extraction. Data extraction categories included: author, year of publication, source of publication (e.g., journal name), where the study was conducted, stated study aims, interventions assessed, study design, study population and sample size, inclusion and exclusion criteria, follow‐up period, outcomes assessed, key findings (relevant to the study questions) and detailed quantitative findings.

### Data synthesis

Data were collated and summarized utilizing descriptive and thematic analysis. Descriptive analysis allowed a description of studies reviewed, including the number of studies, study designs utilized, populations analysed and the source of the works. Thematic analysis was carried out using an inductive model where the data were analysed without a priori conceptions or specific hypotheses to test [6]. This allowed themes to emerge organically during the review process based on recurring patterns and insights from the reviewers and discussions with the wider research team.

## RESULTS AND DISCUSSION

### Descriptive analysis

A flow diagram following PRISMA‐ScR conventions was created and reports the output from the study search and selection process (Figure 1). The initial search identified 156 potentially eligible articles from the Directory of Open Access Journals, Scopus, ProQuest Central, ClinicalTrials.gov, PubMed databases, Google search of grey literature as well as an examination of the citations of relevant articles, and two articles recommended by experts in the area. Of these, 42 were found to be duplicates and were removed. The abstracts of 114 articles were screened, with 89 not meeting the inclusion criteria. A full‐text review was conducted on 25 eligible articles; 0 were excluded. Data extraction was conducted from 25 included articles.

### Characteristics of studies

Of the included studies, there were no randomized controlled trials, systematic reviews or meta‐analyses. There were 18 (72%) retrospective studies that included retrospective cohort studies, comparative studies and registry analyses [7, 8, 9, 13, 21, 24, 25, 26, 31, 33, 34, 37, 42, 43, 47, 53, 56, 62], 4 (16%) prospective studies, all of which were cohort studies [3, 11, 22, 52], and there were 3 (12%) biomechanical cadaveric studies [18, 19, 50].

Seventeen studies reported the age of patients involved, with a mean age across all studies of 67.8 years. Across all included studies reporting demographic information, the mean proportion of female patients was 56.8%. The mean body mass index was 30.0, calculated from the 15 studies reporting this. Of the 14 studies reporting the knee pathology involved, 12 studied only patients with osteoarthritis, and 2 studies also involved low numbers of patients with inflammatory arthritis. The median number of study participants undergoing VRAS knee arthroplasty (when excluding cadaveric studies) was 121. The majority of articles were published in the last 2 years, with only five being published before 2024. Articles originated from eight different nations, with a notable lack of publications from Europe, with North America having 48%, India 20%, Oceania 12%, the Middle East 8%, Asia 8% and Europe 4%.

The level of evidence in studies was varied, with 1 Level II study, 14 Level III studies and 7 Level IV studies. Any conflict of interest relevant to VRAS was also assessed. This area was relatively nuanced, with a wide degree of conflicts present, ranging from paid employees of the manufacturer producing the publications to speaker or consultant fees paid to one or more authors of other papers. All articles that have possible influence from a conflict of interest have been noted, and with this, it was seen that 16 of the 25 papers noted some form of conflict related to the manufacturer of the VRAS device. Table 1 summarizes the articles included with their self‐reported conclusions and includes their assessed conflict of interests as well as their level of evidence.

**Table 1:** Summary of articles reviewed.

Author	Year	Sample size	Follow‐up period	Level of evidence	Conflict of interests	Study overview	Study conclusions	Limitations
Londhe	2025	60	N/A	IV	Nil	Review of the first 60 VRAS cases at a single centre.	Learning curve plateaued after 15 cases. Imageless VRAS system appeared to enable workflow efficiency.	Retrospective, single‐centre, single‐team design. No long‐term data included. No account for room set‐up time.
Alton	2025	100	1 year	II	Yes	1:1 multicentre randomized prospective cohort study comparing VRAS with conventional TKA, including information on VRAS adoption‐phase.	VRAS TKA achieved significantly greater mechanical alignment accuracy than manual TKA FJS and pain better with VRAS than manual TKA at 12 weeks, but otherwise, no significant differences in outcomes. Less adverse events in the VRAS cohort.	Short‐term follow‐up period. Not powered for PROM differences.
Daxini	2024	101	3 months	IV	Nil	Review of the first 101 VRAS cases at a single centre.	100% accuracy in achieving coronal alignment within ±3° of neutral using VRAS. Tourniquet time decreased significantly with experience. No difference in deformity correction or OKS between early and later cases.	Short‐term follow‐up period. Retrospective, single‐surgeon design, limiting generalizability.
Pagan	2024	80	N/A	III	Yes	Review of the first 80 VRAS cases at a single centre and compared the operating time from these to the last 80 conventional TKA cases carried out at their centre.	VRAS reached surgical time proficiency after nine cases and was optimized from case 53. Final VRAS operative times became statistically similar to manual knee arthroplasty; however were still slightly longer in duration.	Single‐surgeon, single‐centre, high‐volume experience may not generalize to all settings. No outcome measures included No account for room set‐up time.
Huang	2024	827	90 days	III	Yes	A retrospective cohort study using a large healthcare database to review 90‐day outcomes from patients undergoing VRAS compared to other robotic‐assisted technologies.	VRAS had significantly lower 90‐day revisit rates than other robotic systems. Total 90‐day costs were significantly lower for VRAS. Operating room time and revision rates were similar between VRAS and other robotic‐assisted technologies.	Retrospective database design limits causality and relies on administrative coding accuracy. Short‐term follow‐up period. No cost‐benefit analysis or deep economic analysis as they were unable to quantify the cost‐benefit of each technology.
Huang	2024	866	90 days	III	Yes	A retrospective cohort study using a large healthcare database to review 90‐day outcomes from patients undergoing VRAS compared to conventional TKA.	Hospital revisits were significantly lower in the VRAS group. VRAS procedures had longer operating room time than manual (138 vs. 134 min). VRAS group had a slightly shorter length of stay. 90‐day revision rate was similar (0.09% VRAS and 0.18% manual). Overall, 90‐day all‐cause and 90‐day knee‐related costs were similar.	Retrospective design with reliance on administrative data. Small VRAS sample size relative to the manual group. Unmeasured confounders (e.g., surgeon skill, patient socioeconomic status). No thorough economic analysis assessing cost‐benefit of each technology.
Rajasekaran	2024	200	6–18 months	III	Nil	A radiographic and clinical outcomes comparative study between VRAS and conventional TKA at a single centre.	Radiographic accuracy was significantly better in the robotic group, with fewer alignment outliers. Functional outcomes improved significantly in both groups, but no significant difference between the robotic and conventional groups. VRAS added around 23 minutes to theatre time on average.	Non‐randomized, retrospective design, subject to selection bias. Single‐centre design. Short‐term follow‐up period. No evaluation of cost‐effectiveness or learning curve implications.
Morrisey	2023	66	6 months	III	Yes	A retrospective comparative study between VRAS and conventional TKA at a single centre.	Robotic surgery times equalized with traditional after two cases. No differences in pain, ROM at 6 weeks, assistive device use, or complications. Only difference was an improved 6‐month flexion in the VRAS group, otherwise no difference was identified.	Retrospective, single‐surgeon design limits generalizability and introduces selection bias. No randomization or long‐term outcome data. Not appropriately powered to highlight differences in PROMs.
Clatworthy	2022	65	1 year	III	Yes	Two separate comparative studies between VRAS and navigation are reported in this article, with one reviewing 6‐week outcomes and the other reporting 1‐year outcomes with a lower sample size.	Improved early pain and function scores at discharge and 6 weeks with VRAS versus navigation. Time‐neutral surgery is achieved after the initial learning curve.	Small sample size for VRAS group. No information on patient selection for each group. Does not have the power to substantiate the improved 1‐year PROMs claimed. Potential author bias (developer of the technique; disclosed consultancy with DePuy Synthes).
Doan	2022	40	N/A	N/A	Yes	A cadaveric study comparing VRAS with conventional arthroplasty	VRAS significantly improved implant and resection alignment accuracy versus conventional instrumentation. Outliers (errors >3°) were fewer in the robotic group across all angular alignment metrics	Cadaveric model lacks soft tissue balance and real clinical loading conditions, limiting clinical applicability 33% of specimens lacked knee arthroplasty‐grade cartilage degeneration, not fully representative.
Benkovich	2025	241	1 month	IV	Nil	A retrospective study reviewing patients with varus or valgus deformity who underwent VRAS TKA and assessing their pre‐ to post‐operative alignment change.	VRAS improved radiographic alignment in both varus and valgus CPAK phenotypes. Valgus knees showed better post‐operative alignment accuracy and required less tibial resection.	Retrospective single‐centre design. No control/comparative group. A number of CPAK groups are excluded, limiting generalizability. May not be powered to draw some of the conclusions Short‐term outcomes.
Bourgeault‐Gagnon	2024	225	N/A	III	Yes	A comparative cohort study assessing differences in polyethylene thickness implanted between VRAS, navigation, and conventional TKA.	VRAS showed greater accuracy and consistency in polyethylene insert thickness versus manual or navigation techniques. Polyethylene thickness outliers (≥ 9 mm) were rare in VRAS compared to navigation and manual techniques Learning curve was short—consistent performance achieved after 30 cases.	Single‐surgeon and single‐implant design limits generalizability. Non‐contemporaneous group assignment may be biased due to evolving surgical skill. No functional or long‐term clinical outcomes reported. Unclear what degrees of deformity were corrected between groups.
Hallak	2025	173	7–9 months	IV	Yes	A prospective study assessing outcomes from patients undergoing restricted inverse kinematic alignment TKA using VRAS.	VRAS with restricted inverse kinematic alignment preserved native knee alignment in most varus knees and neutralized valgus knees. Robotic‐assisted tibia‐first technique showed good reproducibility of CPAK Types IV and V.	No outcome data. Limited generalizability due to single‐centre design. No control group or comparison to other alignment techniques.
Londhe	2024	50	N/A	III	Nil	A study comparing the predicted implant sizes and the definitive implant sizes used between VRAS and conventional techniques.	92% accuracy for exact femoral component size and 100% within one size using VRAS Significantly outperformed conventional sizing accuracy.	Single‐centre, single‐team design. Retrospective approach with historical controls, not randomized or contemporaneous. Patient factors not fully accounted for. No clear description of how conventional sizing prediction was undertaken, and no description of any benefit from the robotic system being more accurate at this prediction if endpoints were similar.
Ho	2025	101	6 weeks for function 12 months for PROMs	III	Yes	A retrospective propensity score‐matched study comparing VRAS TKA with navigated TKA.	VRAS resulted in a significantly shorter navigation time than computer‐assisted surgery. Early clinical outcomes were comparable between VRAS and computer‐assisted surgery groups. No major differences in complications or satisfaction between the groups.	Retrospective design and limited sample size. Only navigation time analysed. Long‐term outcomes and implant. survivorship remain unaddressed. No comparison to manual system.
Spitzer	2024	553	1 year	III	Yes	A retrospective database review of outcome scores following conventional TKA compared with VRAS TKA.	VRAS patients had better KSFS scores at early follow‐up and at 1 year.	Short‐term follow‐up period. Retrospective analysis with registry‐derived data. Inconsistent KSS alignment data prevented full score comparison.
Doan	2022	18	N/A	N/A	Yes	A cadaveric study comparing VRAS with navigated arthroplasty.	VRAS showed significantly better resection accuracy versus navigation and conventional instrumentation. Navigation was more accurate than conventional instruments in some planes but inferior to VRAS overall. Saw being robotically positioned and stabilized, which avoids manual errors during cut execution.	Cadaveric setting: results may not directly translate to clinical outcomes. Limited sample size.
Zheng	2024	159	N/A	IV	Nil	A retrospective review of a national adverse events database comparing different robotic‐assistance technologies.	Mechanical failure most common issue. Lowest proportion of soft tissue damage was reported from any robotic assistance system.	Underreporting and reporting bias are likely due to the voluntary nature of database. Inconsistent report quality, making it difficult to draw causative or comparative conclusions. New systems have disproportionately low numbers of reports. No information on case specifics for any events, with many confounders.
Soundarrajan	2025	14	8–25 months	IV	Nil	A prospective cohort study following a group of patients with significant extra‐articular deformity who underwent TKA using VRAS.	VRAS TKA using functional alignment successfully corrected severe extra‐articular deformities without osteotomy. Functional outcomes improved.	Small sample size. No control group to compare robotic versus conventional correction. Short‐term follow‐up period.
Singh	2021	40	N/A	N/A	Yes	A cadaveric study assessing accuracy of VRAS TKA compared with conventional TKA.	VRAS showed significantly greater accuracy in alignment than conventional instruments. No VRAS cases had soft tissue compromise, while 10% of conventional arthroplasty cases showed minor posterior cruciate or capsule injury.	Cadaveric study only—lacks real‐world surgical dynamics or patient outcomes. Surgeon bias possible, as same group assessed soft tissue outcomes.
Brelin	2025	25	1 year	IV	Yes	A prospective observational study reviewing the learning curve during VRAS adoption as well as early patient outcomes.	Proficiency was reached after five cases overall (4–7 cases for each stage of procedure to reach proficiency). Stage times significantly decreased in the proficient phase, particularly for robotic‐assisted bone cutting. Patient outcomes are similar throughout learning curve.	Single surgeon, single centre design. No comparison to manual TKA. Small sample size.
Girod	2025	280	N/A	III	Nil	A radiographic assessment of accuracy between conventional TKA and VRAS TKA using a deep learning model.	VRAS reduced mechanical axis alignment outliers in the sagittal plane and improved accuracy. No significant difference in early outcomes between manual and VRAS TKA.	Single‐centre design. Only considers one axis of alignment. The deep learning model used was not validated.
Chen	2025	65	6 months	III	Nil	A retrospective propensity score‐matched study comparing VRAS TKA with conventional TKA.	VRAS had significantly shorter surgical duration, improved ambulation distance, and shorter length of stay relative to conventional TKA. VRAS displayed a trend towards higher SF‑36 outcome measures, and a larger proportion of patients achieved SF‑36 bodily pain MCID. More patients reported satisfaction and expectation fulfilment, but this was non‑significant.	Single‐centre design. No longer‐term outcome data. Non‐randomized, retrospective design, subject to selection bias. Comparison group data were collected at different time points to VRAS data, and therefore differences may be affected by wider service changes.
Severson	2025	215	In‐patient	III	Yes	A retrospective cohort study assessing opiate use and length of stay between VRAS TKA and manual TKA.	Manual surgery patients used nearly twice as much pain medication and had longer mean length of stay.	Single surgeon, single centre design. Non‐randomized, retrospective design, subject to selection bias. Comparison group data were collected at different time points to VRAS data, and therefore differences may be affected by wider service changes.
Le Guen	2025	149	N/A	III	Yes	A retrospective comparative study assessing learning curve for VRAS TKA and comparing operative times with navigated TKA.	The learning curve was reached after performing between 4 and 11. The robotic operating time was 57.1 min compared to 54.1 min with navigation. Use of the robot, surgeon, use of a posterior‐stabilized implant and varus of more than 10° were independent factors associated with extended operative time.	Single‐centre. Non‐randomized, retrospective design, subject to selection bias. No clinical outcome assessments or assessments of safety in learning curve.

*Note*: Conflict of interest has been marked as ‘Yes’ where authors may have declared conflicts of interest related to DePuy Synthes/Johnson & Johnson MedTech, or where authors involved in the work are paid employees of those companies, the degree of conflict is not considered.

Abbreviations: CPAK, Coronal Plane Alignment of the Knee; FJS, Forgotten Joint Score; KSFS, Knee Society Function Score; MCID, minimal clinically important difference; N/A, not applicable; OKS, Oxford Knee Score; PROM, patient‐reported outcome measure; ROM, range of motion; SF‑36, Short Form (36) Health Survey; TKA, total knee arthroplasty; VRAS, VELYS Robotic‐Assisted Solution.

### Thematic analysis

Thematic analysis was carried out using an inductive model, allowing themes to emerge organically during the review process based on recurring patterns and insights from the reviewers and discussions with the wider research team. Following discussion, the following six key themes were identified: implant position and alignment (*n* = 11), clinical outcomes and patient‐reported outcome measures (PROMs) (*n* = 12), surgical efficiency and workflow (*n* = 14), safety and complication profile (*n* = 9), soft tissue or bone preservation (*n* = 4) and versatility and complex case use (*n* = 3). The majority of studies addressed two or more themes.

#### Implant position and alignment

A critical role of robotic assistance in arthroplasty surgery is to minimize human error and maximize operative accuracy [45]. There remains debate around what the target for implant positioning should be, and various alignment philosophies exist [44]. Irrespective of these competing philosophies, robotic systems allow surgeons to make more accurate bone resections and achieve more precise implant positioning in accordance with the operative plan [14].

Cadaveric studies have compared conventional (manually instrumented) knee arthroplasty to VRAS robot‐assisted knee arthroplasty and have shown that VRAS knee arthroplasties were more accurate in implant positioning relative to controls using manual instruments, with fewer outliers in all measures [19, 50]. The final implant alignment, coronal and sagittal femoral alignment as well as tibial coronal alignment, were all seen to be more accurate in VRAS cases. These studies both involved small numbers, and of course, although useful to show proof of concept, cadaveric studies do not truly replicate normal use. Doan et al. compared VRAS knee arthroplasty to an already established image‐free knee arthroplasty navigation software (Brainlab Knee3) [18]. This study noted that although the Brainlab navigation software reduced mean absolute errors in resection relative to manual knee arthroplasty, it was still less accurate than the cuts made using the VRAS system. Again, although useful to highlight that the VRAS system can perform accurately, this information, often small improvements in accuracy toward a pre‐defined target relative to other technologies, does not inform surgical practice, as there may be no significant benefit to patients from these differences.

There are several studies evaluating the effect of VRAS use on implant alignment and positioning in living patients [3, 13, 21, 43]. Daxini et al. reviewed 101 consecutive patients who underwent VRAS knee arthroplasty. In all cases, the planned coronal plane alignment was achieved. There was also no significant difference in the accuracy of coronal or sagittal plane deformity correction, or magnitude of range of motion restoration, between the first and second group of 50 cases, which implies that the VRAS system provides effective guidance independent of any learning curve phenomenon. This study supports the conclusions of cadaveric studies, demonstrating their reproducibility in vivo, but it is limited in that it is a small single‐surgeon series, and there is no comparison made to prior manual cases at the same institution.

Rajasekaran et al. and Alton et al. compared manual TKA and robot‐assisted TKA using VRAS; the former was a retrospective cohort study, and the latter was a prospective, multicentre matched case‐control study. Rajasekaran et al found the manual group had significantly higher numbers of outliers in the post‐operative HKA angle, lateral distal femoral angle (LDFA), and tibial slope when compared to VRAS. This study was limited by a small sample size, which increases the risk of a Type I error, especially when comparing subgroups on a single radiographic measure. Alton et al found no significant difference in the mean absolute error of the hip–knee–ankle (HKA) angle between the VRAS and manual group, but the accuracy of femoral and tibial implant angles was better with VRAS; this was statistically significant, but the magnitude of the difference was small. Girod et al. compared variability in radiographic outcomes, and their findings supported those of other studies; VRAS‐assisted cases demonstrated bony resections (in this case, a tibial slope) that were closer to a planned target than the manual cases. They also reported less variability and outliers in bony resections in the VRAS group, supporting the previous reports of increased precision when using this robot‐assisted system [21].

Other studies have evaluated alignment and positioning in specific deformity patterns and using differing alignment philosophies [22, 56]. Benkovich et al. showed significant improvements in both varus and valgus groups in extension, extension/flexion gaps, joint line obliquity and HKA angle, even in those with severe pre‐operative deformity [56]. However, this study was limited by being restrictive in recruitment, with no neutral HKA patients included. The study also has a small sample size and no comparison with conventional instrumentation. Hallak et al. evaluated the accuracy of VRAS in the context of TKA, applying restricted inverse kinematic alignment philosophy and demonstrated good accuracy in preserving HKA angle and achieving the planned adjustment to joint line obliquity in both varus and valgus knees [22, 59]. This study was limited by a small size and no comparison group. These studies suggest that VRAS facilitates accurate and precise implant positioning in a range of knee morphologies and contexts, and that this may be better than manual instrumentation. However, the majority of these studies have small samples and are from single centres with early adopters.

#### Clinical and functional outcomes

Numerous studies have addressed clinical outcomes, including hospital revisits or revisions, infections, post‐operative mobility, instability and mortality, following VRAS knee arthroplasty [3, 9, 11, 13, 22, 24, 25, 26, 37, 43, 47, 53]. There is broad agreement that, in comparison to manual knee arthroplasty, there is no relevant difference in complication rates, use of mobility aids post‐operatively, incidence of infection and acute revision rates are low. Alton et al. concluded that adverse events are significantly less common in VRAS patients relative to conventional instrument patients, but this study included only a small number of patients, and the rate of observed adverse events, including infection, was higher than population registry levels in the conventional group [30]. Huang et al. reported that VRAS cases had a lower rate of hospital revisits and readmissions than manual arthroplasty patients, and that the rates were lower than for other robotic assistance systems. Although the study controlled for confounders such as smoking status, age and comorbidities, a short follow‐up period of only 90 days limits the value of conclusions drawn [25, 26].

The literature is typically limited by poor methodological robustness, particularly a lack of control groups, small sample sizes and a high risk of bias. There is also a lack of evidence relating to longer‐term outcomes, with most studies focusing on the early postoperative period. Despite its limitations, the literature relating to clinical outcomes is useful insofar as there appears to be no significant increase in intraoperative or early postoperative complications versus conventional and established techniques. More research is required in larger sample sizes to demonstrate any significant benefit here, and longer‐term studies are required to evaluate implant survivorship and revision risk.

Several studies have evaluated the effect of VRAS on functional outcomes using common patient‐reported outcome measures (PROMs) [3, 9, 11, 22, 24, 37, 43, 47, 53]. There was agreement between studies that compared functional outcomes between VRAS and manual arthroplasty that VRAS was associated with better early pain and PROMs scores, while range of motion was similar between techniques [3, 9, 11, 22, 24, 37, 43, 47, 53]. However, Chen et al. did show improved pain scores and patient satisfaction out to 6 months post‐operatively in VRAS cases compared to manual arthroplasty, showing some variety in outcomes from different studies, but it should be noted that the differences in these outcomes between groups were still small, with both conventional and VRAS groups showing significant improvement [9]. The improvement in acute pain outcomes does appear to be reflected in many studies, with Severson et al. showing that actual use of opiate analgesia was significantly reduced in the VRAS group, compared to the conventional group [47]. These conclusions are heavily caveated that many of the studies demonstrated no significant difference between outcome measures according to technique, which may be a function of small sample size, small effect size, ceiling effects with existing PROMs scores or the differences arising by chance.

One study assessed the effect on outcomes of the learning curve associated with introducing VRAS into a knee arthroplasty practice. There was a significant reduction in tourniquet time between the surgeon's first 50 cases and the second 50 cases. There was no significant difference in PROMs for cases performed early in the learning curve when compared to cases performed later with proficiency achieved [13]. This area requires further investigation with both greater control of confounding as well as longer follow‐up.

Another limitation of the current literature is that the methodologies applied are inconsistent, with studies evaluating a range of different outcome measures at various timepoints and in heterogeneous study populations.

#### Surgical efficiency and economics

Surgical efficiency is an important factor when considering new surgical technologies [28]. Significant reductions in surgical efficiency due to VRAS would not be acceptable in cases where no benefit could be seen relative to other methods; however, there may still be value even if significant reductions were seen, for example, in complex cases where benefit could be seen.

Several studies evaluated surgical efficiency and reported that VRAS was associated with a longer surgical time, with two studies comparing operative times between VRAS and conventional manual TKA reporting increases of 7–23 min [13, 26, 31, 34, 42, 43]. However, other studies reported no significant difference, or even improvements in surgical time using VRAS when compared to established manual and navigated manual systems, but these were often carried out in high‐volume academic centres, or in some cases were carried out by developers of the technology with close links to industry [9, 11, 24, 25, 37]. Despite this heterogeneity in findings related to surgical time (i.e., the duration of the operation itself), there was agreement across the studies that total operating room time for each case (i.e., the duration of the operation plus setup and turnaround time) was increased relative to manual cases, but the magnitude of increase reported varied from 4 to 23 min [31, 42, 43]. This likely reflects differences in surgical workflow, but also that the studies were carried out during the study centre's adoption of VRAS. There was agreement amongst the articles that surgical time and total operating room time reduced as teams became more familiar with the technology, and in some cases became close to time‐neutral with manual knee arthroplasty. Huang et al. compared operating room times for VRAS with other robotic assistance devices and found no significant difference [25].

Although the evidence is relatively immature, these early studies suggest that VRAS use is associated with a longer time in the operating room per case, but that this effect is mitigated as surgical teams gain familiarity with the system.

A limitation of the literature pertaining to surgical efficiency is that the studies were predominantly conducted in specialized teaching centres, and with teams that may have been relatively familiar with robotic arthroplasty systems. Further studies in different types of hospital and surgical centre settings would add value.

This conclusion is supported by the findings of studies that have evaluated the learning curve associated with VRAS adoption. Two studies used cumulative sum charts to identify inflection points in surgical performance (based on surgical time) and found that surgeons needed approximately nine cases to reach proficiency in one study, with proficiency gained between four and eleven cases in another study, with optimized performance achieved after around 50 cases [31, 42]. These findings have been replicated in other studies, with Brelin et al. suggesting a learning curve of five cases with surgical time reaching a plateau after around 15 cases [8, 34]. These studies should be replicated with a greater size and diversity of surgeons and surgical settings to determine the generalizability of these findings. Further, it is important to highlight that these studies purporting to evaluate ‘learning curve’ used surgical time as a surrogate marker of proficiency, and more research is required to evaluate the learning curve related to the quality and safety of procedures.

Length of hospital stay is an important determinant of surgical efficiency, cost and productivity and is important in both private and socialized healthcare settings. A large registry study conducted on a population level and involving a range of healthcare contexts found that the length of stay in hospital was shorter for VRAS patients than for manual arthroplasty patients (3.1 vs. 3.6 days) [26]. This study was limited by the low number of VRAS cases that were included, as well as the small number of centres in which they were carried out, and the difference may reflect local practice rather than the effect of the robot‐assisted system. Two other studies also assessed hospital length of stay, in smaller cohorts with Chen et al. showing a reduction in length of stay between VRAS and conventional systems from 3.66 to 2.48 days, and Severson et al. noting a more marginal improvement from 1.2 to 1.0 days' length of stay [9, 47]. Despite some variety in length of stay noted following VRAS, these findings of shorter stays are supported by an established body of evidence from other systems that demonstrates robot‐assisted arthroplasty is associated with better early postoperative pain and function, and a shorter hospital stay [28, 61].

Two studies assessed the cost of VRAS; one used manual knee arthroplasty as a comparator, and the other compared it with other robot‐assisted systems [25, 26]. Overall, the 90‐day cost of VRAS was approximately $400 more expensive per case than knee arthroplasty with conventional manual instrumentation. However, it was more than $1800 less expensive per case than other robotic systems [25, 26]. A significant cost saving versus other robotic devices is the lack of requirement for a pre‐operative planning CT scan and the associated radiology report, which is also associated with an extra hospital visit and a built‐in lead time before surgery. These studies were carried out by the group using the same regional database, with relatively low numbers of VRAS cases. They are also limited in that they only considered the index cost of the surgical procedure and any direct follow‐up, and did not consider ancillary costs such as medication or walking aids, both of which may be lower with robot‐assisted arthroplasty [28, 61]. They also did not consider the cost of acquiring and maintaining the robotic system itself. Two studies highlighted the ability of VRAS to facilitate anticipation of correct component sizes, which may save time associated with waiting for component retrieval and preparation, and reduce the economic and ecological cost of larger surgical trays that require more capital equipment and higher sterilization service use [7, 33].

There is a lack of genuine economic analysis that includes the economic exposure of the institution, as well as the costs and savings associated with VRAS use across the whole patient journey. Furthermore, longer‐term studies evaluating outcomes and implant survivorship will be required before conclusions can be drawn about potential cost savings associated with improved outcomes and/or reduced revision rates.

There is little current evidence to support any improvement in surgical efficiency that VRAS may offer, and similarly, there remains a lack of detailed economic analysis to show the true cost associated with the technology.

#### Safety and complication profile

Safety is paramount when introducing any new surgical technology, and an evaluation of patient perceptions of robot‐assisted arthroplasty identified that people have concerns about these systems causing harm [1].

This review of the VRAS literature found that surgical complications were rare, and comparative studies between VRAS and other technology‐assisted or manually‐instrumented knee arthroplasty demonstrated no evidence of additional harm associated with VRAS [3, 11, 22, 24, 25, 26, 37, 62], and some studies suggested that adverse events were less common in VRAS groups [3, 24]. These studies only evaluated intraoperative and early postoperative safety and were limited by small sample sizes. Zheng et al. reviewed the prospectively collected USA FDA Manufacturer and User Facility Device Experience database for complications involving all orthopaedic robotic devices currently on market in the USA, and found that of the 839 reported adverse events, VRAS was implicated in 159 cases (19%) [62]. The most common adverse events were mechanical failure of the robot (*n* = 69, 43%) and inappropriate bone resection (*n* = 49, 31%). This reflects a higher number of reported inappropriate bone resection events relative to other robotic devices, but the findings are difficult to interpret with any reliability because of the methodological limitations: there was no consistent practice for the definition and reporting of adverse events, meaning events may be under/over‐reported; no data are available regarding the clinical significance of each event; it was not possible to evaluate the cause or context of reported events, and no denominator was available for the total number of cases conducted using each system.

Further clinical and registry‐based studies should evaluate the short‐ and long‐term safety of VRAS in line with the standard practice of evaluating any novel surgical technology.

#### Soft tissue and bone preservation

Modern principles of knee arthroplasty recognize the need to preserve the soft tissues around the joint, which includes reducing unintentional iatrogenic injury and the reliance on soft tissue ‘releases’ to produce a balanced prosthetic joint [10, 27, 36]. Soft tissue damage may be associated with: pain and slower early post‐operative recovery; impaired kinematics, function and patient satisfaction; poorer implant survivorship and, in cases of severe injury, the necessity for on‐table or late conversion to highly constrained components [10, 27, 29, 36, 58].

The VRAS is intended to facilitate total or partial knee arthroplasty whilst preserving soft tissue and bone by using technology to evaluate real‐time and anticipated gap and soft tissue balance, bone resection requirements, limb and joint line alignment and implant position [13]. The VRAS functional alignment method aims to restore the native knee kinematics while reducing the need for soft tissue releases [16, 48]. The VRAS mechanical alignment method aims to achieve a neutral limb alignment and joint line obliquity with accurate and minimal bone resection [16, 48].

Existing studies support the manufacturer's claims that VRAS reduces the need for soft tissue releases to achieve a balanced knee and suggest that this is likely to be associated with improved early functional outcomes, pain score and recovery timeframes [54]. The degree to which this benefit can be attributed to the VRAS system specifically (rather than the use of a functional alignment strategy irrespective of the system used) is not clear. However, it is generally accepted that robot‐assisted arthroplasty technology is necessary to achieve effective functional alignment due to the limitations associated with conventional manual instrumentation [48].

One study compared VRAS, navigation using Brainlab Knee3, and manual techniques and their ability to execute a pre‐operative arthroplasty plan to achieve a balanced ATTUNE TKA using a 5 mm thick polyethylene tibial insert [7]. The authors found that the number of outliers (defined as cases in which a polyethylene liner with thickness >9 mm was required) was significantly lower in the VRAS group, and from this, they inferred that the VRAS system was a more favourable technique for preserving bone and soft tissue. Although these findings are consistent with the findings of other studies that indicate a reduction in ‘outliers’ of alignment and positioning when using robot‐assisted systems, the use of polyethylene liner thickness as a surrogate for quality is of limited value [21, 43].

Unlike the Mako system, which has the largest market share among robot‐assisted arthroplasty systems, VRAS does not currently employ formal haptic boundaries for the protection of soft tissue structures around the knee. Instead, protection of these structures is achieved through visual feedback provided to the surgeon who uses the saw more conventionally (although the resection plane is controlled for accuracy). Data from the FDA adverse events database suggests that VRAS has the lowest frequency of (reported) soft tissue injury amongst the robot‐assisted systems that are monitored, although the limitations of this process are acknowledged above [62]. Singh et al. conducted a cadaveric study that compared VRAS with manual instrumented TKA techniques and found that in all VRAS cases, the soft tissue envelope of the knee was intact, but in the manual instrumentation group, 5% of cases had partial posterior cruciate ligament injuries, and 5% had posteromedial capsule compromise [50].

There is a need for more accurate reporting of soft‐tissue releases required during these studies and linking this to outcomes, as this would help in determining the value of this technology.

#### Versatility and complex arthroplasty

The majority of the VRAS literature evaluated its use in the context of routine primary knee arthroplasty, but three studies consider its role in complex cases [22, 52, 56]. Benkovich et al. reported good accuracy of correction when using VRAS in the context of severe varus and valgus deformity (HKA of −6.96° to 6.05°) [56]. Hallak et al. evaluated the use of VRAS when using a restricted inverse kinematic alignment philosophy and reported that the technology facilitated good accuracy and the effective delivery of the operative strategy [22]. Soundarrajan et al. evaluated the utility of VRAS to facilitate intra‐articular correction of severe extra‐articular deformity using a functional alignment philosophy and reported encouraging results [52]. Although these studies are limited by small sample sizes, varied methodology and case series design, they add value to the literature by demonstrating real‐world applicability and considering use cases that can be generalized to broader arthroplasty practice.

#### Industry influence and conflicts of interest in the current literature

VRAS is a relatively new technology, which still warrants robust investigation to ensure not only long‐term safety of the device, but also to ensure that any investment in the technology provides meaningful benefit to the patients who undergo surgery with the assistance of the device. Currently, the literature available on VRAS may hint at some possible benefits to early function, pain and outcomes, but these benefits are not well‐evidenced based on the current publications, and further work is required to produce more high‐quality work. As well as ensuring that the quality of research into VRAS is of a high standard, we must be cautious to ensure that industry influence on this work is minimized to provide the most valuable results.

Significant attention must be drawn to the influence of industry on the current research landscape related to VRAS. With 64% of articles having some degree of conflict involved related to the manufacturer of the device. The conflicts noted are also often significant, with publications from paid employees of the company making up a not insignificant portion of the current literature. On top of this fact, the papers published outside of the company itself are often published by those who have been involved in the design and development of the device, or are published by those with strong ties to the manufacturer.

The papers in the literature that do not have a conflict of interest are often those small, single‐centre reviews of a number of first cases undertaken with the device, and otherwise, the papers are often just short case series, which seriously limits the conclusions that can be drawn from these non‐conflicted papers. Chen et al. and Rajasekaran et al. represent the only two Level III evidence studies assessing patient outcomes that do not suffer from a conflict of interest [9, 43]. This produces an uneasy situation where the majority of the evidence related to patient outcomes, pain, length of stay, efficiency of use, economics and complications is based on results from studies with serious conflicts of interest related to the technology. Each of the points from the discussion of this paper should be taken in this context and be considered cautiously.

There is a need for non‐conflicted, high‐quality evidence production regarding this device, and any clinical decisions made based on the current literature should be cautiously considered based on the degree of industry influence.

#### Knowledge gaps and research priorities

**Figure 2:**
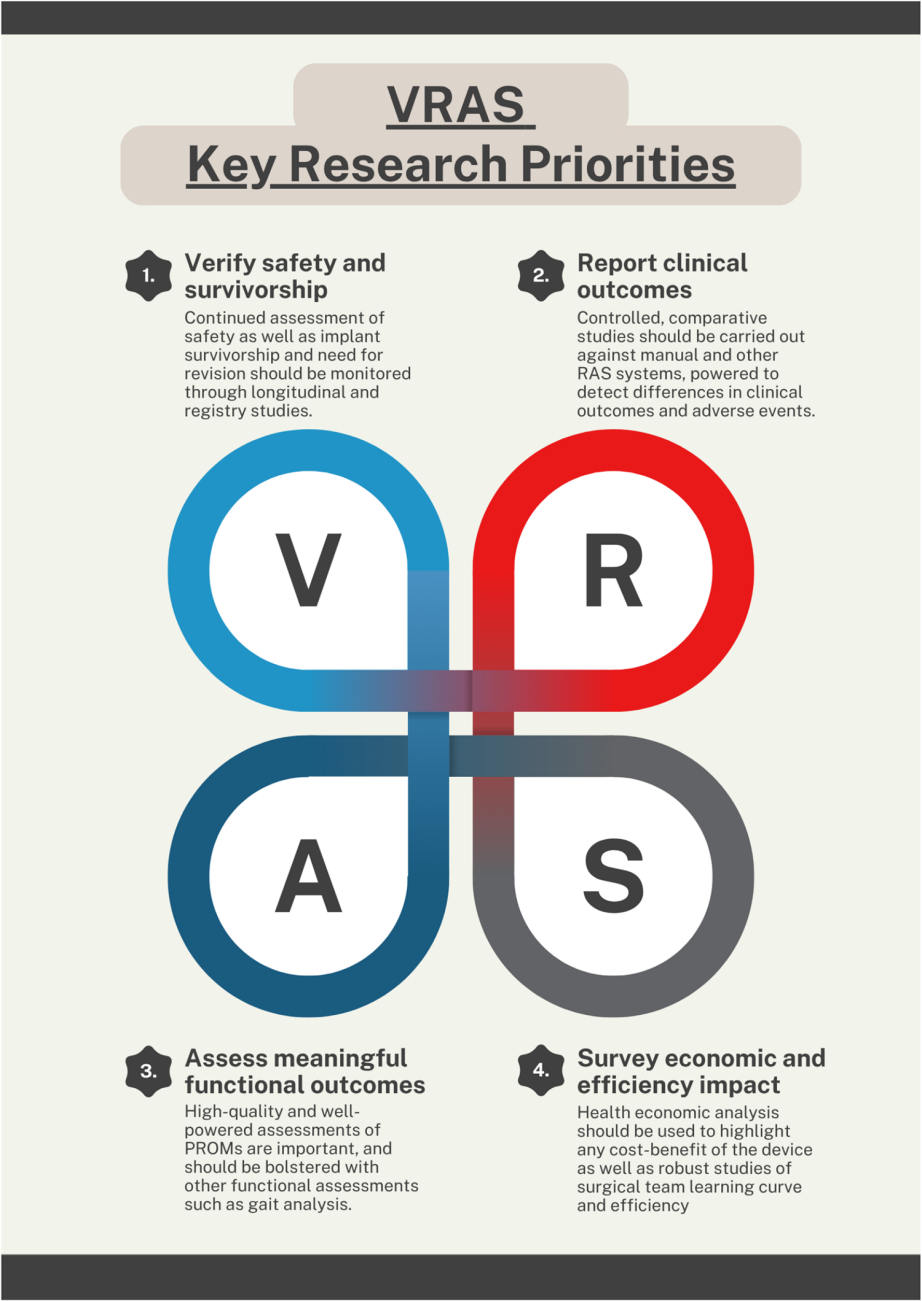
Infographic describing the four key areas of research priority, guided by current gaps in knowledge and demand from the NICE early value assessment. NICE, National Institute for Health and Care Excellence; VRAS, VELYS Robotic‐Assisted Solution.

The overall quality of data is poor, and there remain large and important gaps in knowledge related to the VRAS system. As above, there is also significant industry influence in the current literature, which should be avoided in work into the future. Four key areas were identified to focus future research priorities around VRAS, which are discussed below and represented in Table 2 and Figure 2.

**Table 2:** Table covering key research priorities related to the VRAS system.

Key questions	Research priorities
Is VRAS safe, and do the implants have good survivorship?	Long‐term longitudinal and registry studies to review adverse events and revision rates with registries that not only track the use of a robotic‐assistance device but also specific manufacturer details.
Is there a benefit to clinical outcomes from use of VRAS?	Robust, controlled, comparative studies (both experimental and observational), powered sufficiently to detect important differences in clinical outcomes as well as adverse events, should look at comparing VRAS with manual surgery as well as other RAS systems. These studies should make sure to undertake thorough subgroup analysis.
Is there a benefit to functional outcomes from use of VRAS?	Robust, controlled, comparative studies should be able to detect clinically important differences in PROMs (as subjective outcomes). These studies should be powered to show minimally clinically important differences. Motion analysis laboratory studies may also help to better define functional outcomes (as objective outcomes).
What are the economic and surgical efficiency impacts of using VRAS?	Evaluation of micro‐ and macro‐economics must be undertaken to fully understand the possible cost‐benefit of this technology. Further studies should assess time in theatre, and surgical team workload and learning curve analysis.

Abbreviations: PROM, patient‐reported outcome measure; VRAS, VELYS Robotic‐Assisted Solution.

Firstly, there must be continued monitoring of the safety of this device, with robust follow‐up allowing for identification of any events as well as fully characterizing these, to ensure patient safety is maintained and optimized. There is also the need for longer‐term implant survivorship data, assessing any returns to theatre and rate of any revision surgery. Both of these can be assessed through longer‐term longitudinal and registry studies, and it is important that registries are not only tracking use of a robotic‐assistance device, but specific manufacturer details.

Secondly, the utility of this device must be thoroughly studied through robust, controlled, comparative studies (both experimental and observational). These studies must be powered sufficiently to detect important differences in clinical outcomes as well as adverse events and should look at comparing VRAS with manual surgery as well as other RAS systems. These studies should make sure to undertake thorough subgroup analysis of those with different knee deformity phenotypes to fully understand device utility, as is laid out in the NICE early value assessment document [41].

Thirdly, to bolster the above work on clinical outcomes, high‐quality and well‐powered functional outcomes evaluations must be undertaken. These should also utilize randomized controlled trials where possible and should be able to detect clinically important differences in PROMs (as subjective outcomes). With current PROMs subject to ceiling effects, and possibly not being able to detect enough of a change to be deemed significant but possibly having results that maintain clinical importance, other outcomes should be assessed in comparative studies, and motion analysis laboratory studies may play an important role in this work, particularly for early functional outcomes (objective outcomes) [12].

Finally, health economic analysis and surgical efficiency studies should be carried out utilizing robust methodology. Evaluation of microeconomics (cost per case, cost to institution, surgical efficiency, etc.) and macroeconomics (cost of widespread uptake in networked private or socialized systems, cost/savings associated with services (e.g., radiology) and possible lower revision rate, possibly higher return to work/activity) must be undertaken to fully understand the possible cost‐benefit of this technology. Aside from pure economic analysis, within these further studies should be better understanding of time in theatre, and not just surgeon, but surgical team workload and learning curve analysis.

There remain large gaps in knowledge around the VRAS system, and although the current literature provides some insight, addressing these gaps is essential to fully understand what the system can add and whether it holds enough value for routine implementation in arthroplasty surgery.

## STRENGTHS AND LIMITATIONS

This review has several limitations that should be acknowledged. Firstly, the search was restricted to articles written or translated into English, which may have excluded relevant studies published in other languages. Although the scoping approach allows for a comprehensive mapping of the literature to help guide future research work and give a current overview of work on the topic, it limits the ability to draw definitive conclusions related to current clinical practice and use of VELYS. The heterogeneity in study designs, methodologies and populations across the included articles presented challenges in synthesizing the results. This can complicate direct comparisons and limit the generalizability of specific findings. As has been noted at numerous points, the articles in this review are often influenced by industry, and although this review does well to highlight this issue, and promote care in assessment of the literature, it should be reinforced, that many of the papers referenced do have a degree of conflict involved, and therefore the results from these should be interpreted in that context.

The review also has a number of strengths. These include the fact that this review was undertaken independently of industry involvement and bias and adheres to a robust scoping review methodology. This review is also timely and focussed appropriately for what has been set out in the NICE EVA.

## CONCLUSIONS

This review presents the current literature regarding the use of VRAS in total knee arthroplasty in response to the recently published call for multi‐domain assessment of six different RAS technologies in orthopaedics from NICE. The current literature is relatively limited in number, with twenty‐five papers identified, and the majority of these have some degree of conflict of interest from the device manufacturer. There does appear to be acceptable evidence of improved implant positioning, similar to other robotic systems, as well as a satisfactory safety profile, and there is evidence of increased cost relative to conventional arthroplasty, but a cheaper cost than other robotic systems. However, many of the studies conclude that VRAS is responsible for benefit to patient outcomes and function, as well as improved efficiency in workflow, but with the quality of current studies, it is not possible to robustly confirm these conclusions. Most studies have multiple limitations in their methodology, sample size, patient selection and control for risk of bias. Evidence is largely retrospective or early prospective, with no randomized controlled trials or long‐term data. The overall quality of evidence is poor to support many of the conclusions drawn. The lack of long‐term data or any randomized controlled trials further limits what definitive conclusions can be drawn about this technology. This review identifies critical directions for future research, particularly in investigating patient‐reported outcomes and function, as well as providing more robust research to confirm the safety and cost‐benefit of this technology. High‐quality, randomized studies are required to show benefit from this technology to confirm its role and justify broader clinical adoption.

## AUTHOR CONTRIBUTIONS

**Scott R. Morrison**: Data curation; formal analysis; methodology; project administration; writing—original draft; writing—review and editing. **Andrew J. Hall**: Conceptualization; data curation; formal analysis; writing—original draft; writing—review and editing. **Nick D. Clement**: Conceptualization; supervision; writing—review and editing. **Phil J. Walmsley**: Writing—review and editing. **Christopher Gee**: Writing—review and editing. **Jon V. Clarke**: Conceptualization; supervision; writing—review and editing.

## CONFLICT OF INTEREST STATEMENT

Nick D. Clement has received research support from Stryker and Johnson & Johnson. Phil J. Walmsley is a member of the Editorial Board of *KSSTA* and has received payment for teaching from LINK and Johnson & Johnson. Christopher Gee has received speaker fees, paid research support and paid consultant for Molnlycke, and serves as an unpaid consultant for Stryker. The remaining authors declare no conflict of interest.

## ETHICS STATEMENT

The authors have nothing to report.

## Data Availability

All data are publicly available in the published literature.
